# Poultry farmers’ knowledge, attitude, and practices toward poultry waste management in Bangladesh

**DOI:** 10.14202/vetworld.2023.554-563

**Published:** 2023-03-22

**Authors:** Soshe Ahmed, Mst. I. Z. Moni, Maksuda Begum, Mst. R. Sultana, Aurangazeb Kabir, Md. J. Eqbal, Sunny K. Das, Woli Ullah, Tasmin S. Haque

**Affiliations:** 1 Department of Veterinary and Animal Sciences, University of Rajshahi, Rajshahi, Bangladesh; 2 Department of Poultry Science, Sher-e-Bangla Agricultural University, Dhaka, Bangladesh; 3 Palli Karma Sahayak Foundation, Dhaka, Bangladesh; 4 Department of Anthropology, University of Rajshahi, Rajshahi, Bangladesh

**Keywords:** attitude, knowledge, poultry waste, practice, waste management

## Abstract

**Background and Aim:**

The improper handling of poultry litter and waste poses risks to humans and environment by introducing certain compounds, elements, and pathogenic microorganisms into the surrounding environment and food chain. However, understanding the farmers’ knowledge, attitude, and practices (KAP) could provide insights into the constraints that hinder the appropriate adoption of waste management. Therefore, this study aimed to assess poultry farmers’ KAP regarding waste management issues.

**Materials and Methods:**

A cross-sectional KAP study was conducted with native poultry keepers and small-scale commercial poultry farmers in seven districts of Bangladesh. In the survey, 385 poultry producers were interviewed using validated structured questionnaires through face-to-face interviews to collect the quantitative data in their domiciles.

**Results:**

The overall KAP of farmers regarding poultry waste management issues demonstrated a low level of KAP (p = 0.001). The analysis shows that roughly 5% of farmers have a high level of knowledge of poultry waste management issues, followed by around one-third of respondents having a moderate level of knowledge. Considering the attitude domain, more than one-fifth of native poultry keepers and nearly two-thirds of commercial producers demonstrated a low level of attitude toward poultry waste management. Considering the overall analysis, roughly half of the respondents found a high level of attitude, and over half of the farmers showed a moderate level of attitude toward poultry waste management issues. The analysis showed that the level of good practices for native and commercial poultry production systems is estimated at 77.3% versus 45.9%, respectively, despite the farmers’ lesser knowledge and attitudes toward poultry waste management systems. Overall, analysis showed that nearly 60% and 40% of poultry producers had high and moderate levels, respectively, of good practices in poultry waste management issues.

**Conclusion:**

Analysis of the KAP data shows that farmers had a low level of KAP toward poultry waste management. The result of this study will assist in formulating appropriate strategies and to adopt poultry waste management solutions by poultry farmers to reduce environmental degradation.

## Introduction

The poultry sub-sector in Bangladesh contributes prolifically to the household economy and provides self-employment opportunities with affordable sources of protein [1, 2]. Poultry production in Bangladesh has gained tremendous momentum in the past few decades. At present, 18 grandparent breeder farms in operation, and 206 small, and large-scale listed parent breeder farms produce around 17–18 million day-old chickens weekly [3, 4]. Approximately 60–70 thousand commercial poultry farms produce 40–42 million table eggs daily [3, 4]. The annual commercial poultry feed production is 5.5 million metric tons [4]. The average annual poultry meat consumption is now 6.3 kg/person, which is expected to reach more than 7 kg/person by 2023 [4]. The current investment in the industry is around 350 billion BDT, which is expected to reach 630 billion BDT by 2030 [3]. The total poultry operation in Bangladesh involves about 6 million people, of which approximately 40% are female [3]. The role of small-scale poultry farming is gaining recognition in promoting gender equality and reducing the food and nutrition security of the farming community [5]. Thousands of commercial poultry microenterprises have flourished in Bangladesh. However, most enterprises must follow proper husbandry practices and waste disposal systems. The production and processing of poultry results in manure, bedding material, hatchery wastes, on-farm mortalities, poultry byproducts, processing wastewater, and bio-solids [6–9]. Approximately 1,560,000 metric tons of poultry waste are produced in Bangladesh annually [10], and poultry waste generation reaches up to 68 billion tons globally [11].

Solid waste affects the environment and is a significant environmental concern worldwide and exerts immense pressure on public health and the environment [12–16]. It includes harmful algal blooms, surface, and groundwater quality degradation, soil quality degradation, widespread anoxia, agricultural plant species diversity, and impacts the native vegetation [17, 18]. Pollutants and pathogens in poultry litter that is traditionally linked to environmental degradation include nutrients, protozoan, bacterial load per area of land, viral agents, and chemical residues. In general, air quality can be affected by the aerial emissions of ammonia and atmospheric pollutants from poultry production facilities [19, 20]. In addition, poultry wastes pose human and environmental risks by introducing certain compounds, elements, and pathogenic microorganisms into the surrounding environment and food chain [21–24]. Poultry manure contains nitrogen, phosphorus, and other excreted substances, such as hormones, antibiotics, pathogens, and heavy metals which are introduced through feed [19, 25].

This risk increases with raw poultry manure, particularly poultry litter, a common practice in the production of vegetables [26]. For many years’, landfills have been used to dispose of poultry wastes in many developing countries. However, the organic waste degradation at landfills could result in leaching to contaminate the surface- and groundwater, which is a primary environmental concern [27, 28]. Aerobic composting of waste is a beneficial treatment that results in fewer microbiological contaminations in the manure [29–32]. However, poultry farms and processing activities can cause a local nuisance due to the emission of a pungent smell, creating an unfavorable environment for individuals in the surrounding area [33]. Of the multiple manure-based compounds that generate odor, NH_3_ gas emits an intense and pungent smell. Good farm design and management practices can assist in minimizing the odor from farm sheds [33].

The improper handling of litter and waste from poultry farms may breach the farm biosecurity management and increase the incidence of diseases, consequently affecting public health welfare. There are diverse methods of disposing of poultry-generated waste, including rendering, burial, incineration, feed for livestock, composting, source of energy generation, and fertilizer [6]. In Bangladesh, many farmers, and home gardeners directly apply poultry manure to the soil, which is a significant cause of pathogenic microbial hazards [34]. Dumping also is not a scientific or efficient poultry waste disposal method [35]. Appropriate poultry waste management can alleviate environmental and health hazards by reducing pathogens, including *Escherichia* *coli* and *Salmonella* [36]. Environmental degradation has become a significant concern because of the considerable amounts of poultry waste produced by commercial poultry farming. Proper management and reuse of wastes can influence the circular economy [37, 38]. The proper disposal of poultry and livestock manure is essential for recyclable resources [39–41]. There is increasing pressure as the production frequencies increase with limited arable land available for manure disposal [27, 42]. The ecological damage that occurs from the improper disposal of poultry waste is poorly understood. Solid knowledge is vital to designing an appropriate waste management system [43].

Poultry farming needs to adjust its management practices and processing activities to favor the environment and reduce environmental pollution. It is argued that sustainable poultry waste management solutions and proper strategies should also optimize the socio-economic and ecological aspects. Understanding the farmer’s knowledge, attitude, and practices (KAP) could provide insights into the farmers’ underlying constraints that hinder the appropriate adoption of waste management. Therefore, the study aimed to assess poultry farmers’ KAP regarding waste management issues.

## Materials and Methods

### Ethical approval

This research did not require ethical approval and an animal care and use certificate because no animals were involved in the study. We informed the participants about the research and its objectives in their language. We explained the consent form to the respondents. Finally, we obtained written and signed informed consent from the participants to participate in the research.

### Study period and location

**Figure-1:**
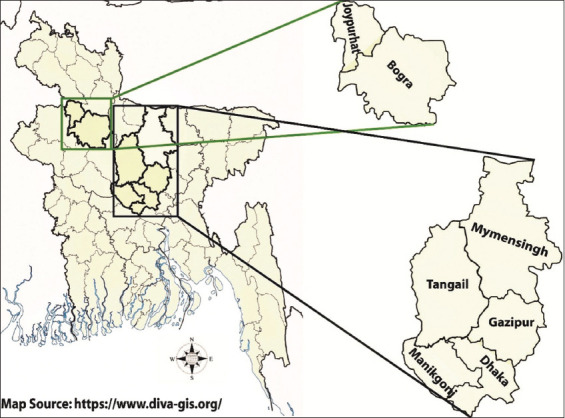
Map of Bangladesh showing knowledge, attitude, and practice study sites [Source: https://www.diva-gis.org/].

The cross-sectional study was conducted from September to November 2021 on native and small-scale commercial poultry farms in seven districts of Bangladesh (Figure-1).

### Knowledge, attitude, and practices study steps

We followed the subsequent steps to conduct the KAP study. Those steps included identifying the topic, selecting the target population and participants, preparing the KAP questions and answer options, scoring the questionnaire, developing and validating the instrument, piloting, data collection and management, and data analysis and interpretation.

### Study design and sampling

A cross-sectional survey study was conducted on native and small-scale commercial poultry farms in seven districts of Bangladesh (Figure-1). We used the following formula to calculate the sample size from the unknown population [44].


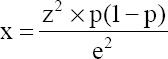


z = 1.96 (value of z for 95% confidence interval)

p = 1-p=50%

e = 0.05 (error level)

The calculated minimum sample size was 384.

In the survey, 385 poultry producers were selected for interview using the convenience sampling technique and included 163 native poultry keepers and 222 small-scale commercial poultry farmers. We performed convenience sampling as it is affordable to gather data from a sizable on-hand population. In addition, it is argued that convenience sampling offers the opportunity to receive specific feedback from individual perspectives. Therefore, despite having fewer representative results over a random sample area, we accomplished the convenience sampling technique to collect information from the poultry farmers from the nearest physical locations of the study areas. The structured questionnaires were personally administered to the farmers through face-to-face interviews to collect the quantitative data in their domiciles. The survey questionnaires used in this study were checked for completeness and consistency before use.

### Development of the KAP questionnaire

We used a KAP questionnaire which comprised two main sections. Section one consisted of six statements concerning the general demographic data and poultry farm enterprise information. Section two assessed the KAP of farmers toward poultry waste management. This section consisted of three sub-sections. The second section consisted of 10 statements intended to evaluate farmers’ knowledge of poultry waste management, 12 statements to evaluate the farmer’s key personal attitudes, and 7 statements to determine the practices of waste management at the farms. The knowledge domain questions consisted of three answer options “correct,” “incorrect,” and “do not know.” The “correct” answer was awarded one mark, and the “incorrect” and “do not know” responses received zero marks. The attitude questions were designed with a five-point Likert scale ranging from “strongly agree” to “strongly disagree” to indicate the degree of agreement toward the statement. Numerical scores 5, 4, 3, 2, and 1 were given to the category “strongly agree,” “agree,” “undecided,” “disagree,” and “strongly disagree,” respectively. We used a four-point Likert scale to assess the practice domain questions comprised of responses “frequently,” “occasionally,” “rarely,” and “never” categories, which were scored as 4, 3, 2, and 1, respectively. The negatively phrased statements in all sections were scored in reverse. We prepared the questionnaires in English, which were translated into Bangla (the national language of Bangladesh). Three academic experts were selected purposely from the Department of Veterinary and Animal Sciences, University of Rajshahi, Bangladesh; they checked and validated the contents and wording of the questionnaire. A study pilot with 20 respondents was employed before actual data collection to test the consistency and reliability of the questionnaire.

### Data management and analysis

Individual respondents’ KAP answers were scored and transformed into a percentage. The total score of each outcome was calculated based on Bloom’s cutoff point [45]. Based on the calculated scores, the level of knowledge of the respondents was categorized into three levels using Bloom’s cutoff point: a low (<60%), moderate (60%–80%), and high (80%–100%) level of knowledge. According to Bloom’s cutoff point, the total attitude scores were also categorized into positive attitude (80%–100%), neutral attitude (60%–80%), and negative attitude (<60%). The collective practice scores of the respondents regarding poultry manure disposal options were also classified into three levels based on Bloom’s cutoff point, that is, poor practice (<60%), fair practice (60%–80%), and good practice (80%–100%).

The respondents’ demographic characteristics and poultry farm-related information were summarized using descriptive statistics. The relative proportions of the respondents’ KAP ratings between the farm types (native chicken keepers and small-scale commercial poultry farms) were analyzed using the Chi-square test as appropriate. p < 0.05 value was set as the statistical significance level and data were analyzed using IBM SPSS Statistics for Windows, Version 24.0. (IBM Corp., Armonk, NY, USA).

## Results

### Demographic and farming-related information

The analysis of demographic and farm-related parameters is summarized in Table-1. The education level attained by most respondents ranged from uneducated to below secondary level. As illustrated in Table-1, less than one-sixth of the respondents could not read and write and around 10% had only completed primary schooling. Just over 5% of poultry farm owners have a graduate degree. Nearly half of the respondents were below 35 years old, about one-third were 36–45 years old, and roughly one-fifth were 46 years or above. The family size of the households among the producers ranged from 1 to 8 members per holding. About half of the households had four or fewer persons per holding. The majority (94.8%) of the families were male-headed. As indicated in Table-1, around one-fourth of the respondents had 3–5 years of poultry farming experience, and nearly one-third had been engaged in poultry farming for 6–10 years. We examined the flock size; almost 40% of the commercial farms kept 1000–2000 birds, and around one-fifth had below 1000 birds/farm.

**Table-1:** Demographic profile and farm-related information (n=385).

Variable	n (%)
Age	
≤35 years	168 (43.6)
36–45 years	125 (32.5)
≥46 years	92 (23.9)
Education	
Uneducated	54 (14.0)
Primary	42 (10.9)
<Secondary	122 (31.6)
Secondary	98 (25.5)
Higher Secondary	48 (12.5)
≥Graduation	21 (5.5)
Household size	
≤Persons	186 (48.3)
5–7 persons	154 (40.0)
≥8 persons	45 (11.7)
Gender of household head	
Male	365 (94.8)
Female	20 (5.2)
Farming experience	
≤2 years	38 (9.8)
3–5 years	107 (27.8)
6–10 years	123 (32.0)
>10 years	117 (30.4)
Number of birds kept	
≤1000	81 (21.0)
1001–2000	150 (39.0)
2001–3000	70 (18.2)
3001–4000	27 (7.0)
4001–5000	19 (4.9)
>5000	38 (9.9)

The knowledge domain items with the corrected answers calculated are displayed in Table-2. A large majority of the farmers do not believe that the emission of gases from poultry production can cause the depletion of the ozone layer. However, more than half of them think that the burning of poultry wastes may cause air pollution. Assessment of their knowledge of water and soil pollution found that below one-fifth of the respondents believed that poultry manure could pollute surrounding waters. At the same time, around one-third assumed that wastewater channels could not potentially contribute to the infection of the water lodged in the poultry setting. However, fewer than half of the respondents correctly answered that the use of untreated manure in the soil was not good, and around half of them categorized that composting poultry manure could increase the availability of nutrients for plants. However, nearly half of the native poultry keepers and more than two-thirds of commercial farmers correctly answered that the long-term storage of poultry manure in open spaces is harmful to the environment. In addition, more than one-third of the respondents identified that poultry wastes are detrimental to human health.

**Table-2:** Frequency of farmers’ knowledge on waste management issues (n=385).

Statement	Number of farmers corrected response	

	Native poultry keeper	Commercial poultry farmer
Burning of poultry manure leads to air pollution	92 (56.4)	156 (70.3)
The emission of gases from poultry production may cause depletion of the ozone layer	19 (11.7)	32 (14.4)
Poultry wastes may cause a risk to human health	143 (87.7)	185 (83.3)
The use of untreated manure in the soil is not good	77 (47.2)	87 (39.1)
Biogas can be generated from poultry manure	73 (44.8)	180 (81.1)
Composting poultry manure can increase the availability of nutrients for plant	80 (49.1)	136 (61.3)
Composting can be the incremental income for the farm	43 (26.4)	155 (69.8)
Poultry manure cannot pollute surrounding water, potentially	38 (23.3)	36 (16.2)
Storage of poultry manure in open space for a long time is harmful to the environment	67 (41.1)	177 (79.7)
Wastewater channels cannot constitute infection in any way	62 (38.0)	40 (18.0)

Only correct answers considered knowledge scores for respondents

Table-3 provides a summary of the attitudes of respondents toward poultry waste management.

**Table-3:** Agreement of farmers’ attitudes toward waste management (n=385).

Statement	Number of farmer corrected response	

	Native poultry keeper	Commercial poultry farmer
Waste management applies from small to large-scale poultry production	73 (44.8)	199 (89.6)
Waste management should be an essential daily activity on the farm	75 (46.0)	181 (81.5)
Poultry manure can be an additional source of income	22 (13.5)	133 (59.9)
Environment pollution can occur from any other sources, so it should not be a matter of concern to me*	90 (55.2)	134 (60.3)
Waste management needs technological interventions, which I do not have	132 (81.0)	164 (73.9)
Only a tiny amount of waste is generated from the farm, so it is not necessary to adopt any waste management system*	85 (52.1)	169 (76.2)
I need to produce bio-manure with poultry wastes as I am also the crop producer	110 (67.5)	176 (79.3)
It is important to me that the poultry should be produced in an environmentally friendly way	108 (66.2)	207 (93.2)
Effective recycling of waste cannot be achieved widely, so it is unnecessary to me*	54 (33.0)	87 (39.2)
If I had more knowledge on this issue, I would integrate environmental considerations into farming practices	115 (70.6)	192 (86.5)
Waste management should be incorporated to reduce environmental degradation	120 (73.6)	196 (88.3)
I am interested in expense a small amount of money to improve waste management on the farm	91 (55.8)	199 (89.6)

Agree includes both strongly agree and agree on responses.

* Statement scores were reversed

Overall, between half to two-thirds of respondents agreed with all the “correct” attitude statements. However, the rate of correct statements was comparatively lower in native poultry keepers than commercial farmers. For example, only 13.5% of native poultry keepers considered that poultry manure can be an additional source of income, while 59.9% of commercial poultry producers agreed with the statement. In assessing the negative attitudes of farmers toward poultry waste management, more than half of the respondents believed that environmental pollution should not be a matter of concern as it can occur from other sources. Furthermore, more than one-third of the respondents incorrectly believed that effective poultry waste recycling cannot be widely achieved. In addition, more than half of the native poultry keepers and two-thirds of the commercial farmers incorrectly believed that the adoption of waste management systems was unnecessary for those who generate a small amount of poultry waste. However, more than half of the native poultry keepers and over three-quarters of commercial producers are interested in spending a small amount of money to improve waste management on their farms.

As shown in Table-4, the highest-scored positive waste management practice statement was that majority (approximately 90%) of the farmers do not use poultry wastes as fish feed, followed by that they do not dump poultry manure on the nearby ground (nearly 90%). Furthermore, more than two-thirds of farmers agreed that they do not prefer to use untreated poultry manure on agricultural land. However, more than half of the native poultry keepers and more than two-thirds of the commercial producers stated that they used poultry wastes for composting. On the other hand, the highest response for harmful practices was that the majority (>90%) of the farmers stated that the poultry manure was sun-dried and burned for cooking. Furthermore, more than three-quarters of respondents agreed that the liquid wastes produced from the poultry settings were connected with the water lodging area.

**Table-4:** Frequency of farmer practices on poultry waste management issues (n=385, Multiple responses allowed).

Statement	Number of farmers corrected response	

	Native poultry keeper	Commercial poultry farmer
I do not dump poultry manure on a nearby ground	132 (80.98)	199 (89.6)
Generation of biogas from poultry manure	1 (00.6)	33 (14.8)
I do not prefer to use poultry manure on agricultural land without any treatment	102 (66.7)	169 (76.1)
I do not use poultry wastes as fish feed	159 (97.5)	194 (87.4)
Liquid wastes are connected with the water lodging area*	123 (75.46)	182 (81.98)
Poultry manure is sun-dried and burned for cooking*	153 (93.87)	215 (96.84)
Poultry wastes are used as composting	88 (53.99)	183 (82.43)

Routine work includes both frequently and occasionally responses.

* Statement scores were reversed

The overall KAP ratings are summarized in Table-5. Regarding the knowledge domain, only 3% of native poultry keepers and nearly 6% of commercial farmers have high level knowledge of poultry waste management. In comparison, almost one-fifth of the native poultry keepers and around 40% of commercial producers were found to have moderate knowledge. On the other hand, more than three-quarters, and over half of the native and commercial poultry farmers, respectively, were found to have a low level of knowledge of poultry waste management issues. The analysis combining native and commercial farms shows that below 5% of farmers has a high level of knowledge on poultry waste management issues, followed by around one-third of respondents with a moderate level of knowledge. Finally, considering the attitude domain, more than one-fifth of native poultry keepers and nearly two-thirds of commercial producers showed a low attitude level toward poultry waste management.

**Table-5:** Overall rating of knowledge, attitude, and practices.

Characteristics	Native poultry keeper	Commercial poultry farmer	Overall	p-value
		
	n (%)	n (%)	n (%)	
Level of Knowledge				
High	3 (1.8)	13 (5.9)	16 (4.15)	0.001
Moderate	30 (18.4)	93 (41.9)	123 (31.94)	
Low	130 (79.8)	116 (52.3)	246 (63.89)
Level of attitude				
High	36 (22.1)	136 (61.3)	172 (44.67)	0.001
Moderate	119 (73.0)	85 (38.3)	204 (52.98)	
Low	8 (4.9)	1 (0.5)	9 (2.33)
Level of practice				
High	126 (77.3)	102 (45.9)	228 (59.22)	0.001
Moderate	35 (21.5)	119 (53.6)	154 (40.00)	
Low	2 (1.2)	1 (0.5)	3 (0.78)	

Chi-square assessed *P* value of the difference in the relative proportions

Interestingly, native poultry keepers were found to have double the moderate attitudes levels toward waste disposal issues than that commercial producers. Furthermore, below 5% of native poultry keepers and <1% of commercial producers were identified to have a low attitude level toward poultry waste management. Considering the overall analysis, nearly half of the respondents demonstrated a high attitude level, and over half had moderate attitude levels toward poultry waste management issues. The analysis in Table-5 shows a high level of good practices for native and commercial poultry production systems (77.3% vs. 45.9%) despite having less knowledge and attitudes toward poultry waste management issues. The moderate practice levels of waste management showed 21.5% and 53.6% for native and commercial poultry producers, respectively. The overall analysis showed that nearly 60% of the poultry producers found had a high level and 40% of the respondents had a moderate level of appropriate poultry waste management practices.

## Discussion

The poultry industry has rapidly expanded over the past few decades, increasing concern about poultry waste disposal systems [46]. Adopting proper poultry waste disposal techniques are crucial to reduce environmental contamination and promoting better farming [47]. The analysis of the KAP questions provides insights from the poultry farmers on their poultry waste management issues. Numerous studies have previously evaluated the atrophying emissions from poultry litter management and gaseous emissions from poultry houses [48–50]. The improper use of poultry manure can result in greenhouse gas production, negatively impacting the environment and may cause the depletion of the ozone layer [19, 51–53]. However, in this study, only a small number of farmers correctly believed that greenhouse gases can deplete the ozone layer. The inadequacy of corrected knowledge results in the farmers may not be aware of ozone layer depletion. The prolonged storage of poultry manure is not scientific and can lead to serious health concerns for animals and humans [35, 54, 55]. Therefore, it is necessary to dispose of poultry wastes in a timely manner and the current study’s findings indicated that over three-quarters of the respondents do not dump poultry manure on the nearby ground. Poultry manure has traditionally been used to improve the soil. However, the over-application of this manure can lead to the contamination of water bodies, spread of pathogens, air pollution, and emission of greenhouse gases [56]. Although poultry manure is a good source of soil nutrients [57, 58], it may contain drug residues, heavy metals, and pathogenic microorganisms harmful to crops, animals, and humans [58–63]. In addition, poor management of poultry manure could promote harmful pathogens in the surrounding soil and water facilities [21, 64]. Therefore, developing efficient alternatives for the disposal of poultry waste that is environmentally safe and secure is prudent. There are different techniques for poultry waste and spent hen’s conversion into a valorized valuable compound. The available techniques are anaerobic digestion of waste to generate biogas and bio-fertilizers; microbial fermentation for bio-alcohols, biodiesel, and bio-hydrogen production; enzymatic hydrolysis to produce biopolymers, biochemical, and bioplastics; carbonization, and activation to obtain bio-sorbents; and incineration, gasification, and pyrolysis to produce bioenergy, bio-char, and bio-oil [65–70]. A few trials have been conducted to transform muscle and collagen protein of spent hens into health-beneficial bioactive peptides [71–76]. Many attempts have been attempted to develop protein or lipid-based biomaterials as potential substitutes for synthetic materials [77–80]. Furthermore, the bioconversion of manure, and organic waste using insects may have a substantial impact. Valorization of manure using insects can be an alternative solution that harmonizes with the circular economy [81]. Using black soldier fly larvae (BSFL) can maximize waste volume reduction and increase insect biomass production. Chicken manure has proved to be a suitable substrate for BSFL [82–83] and can decrease unpleasant odors [84] and lower pathogen proliferation [85]. Nonetheless, the proper storage of poultry litter materials is beneficial and may prevent the contamination of the proximate surface waters and soil. However, in this study, around one-fourth of farmers believe that poultry manure cannot potentially pollute the surrounding water. Another study reported that in Bangladesh, many farmers apply untreated waste to the lands and ponds, which may cause considerable microbial hazards [34]. Likewise, more than three-quarters of farmers mentioned that liquid wastes generated from poultry settings connected with the water lodging area. Furthermore, more than a quarter of the respondents used untreated poultry manure in the soil for crop and vegetable production, which was reported to be undesirable by the study of Kyakuwaire *et al*. [61]. The composting of poultry litter is generally a simple natural biological process of converting poultry litter into odorless manure, adding nutrients to the soil, and reducing pathogens by increasing the beneficial microbes [86–88]. However, the study showed that only around half of the native poultry keepers and more than three-quarters of commercial farmers are involved in the composting of poultry wastes. Poultry manure can be converted into bioenergy [89–92], which may be used as an on-farm and household energy source with socio-economic and environmental benefits [46]. However, despite having numerous opportunities, Bangladesh is lagging in the generation of biogas at a large-scale. The current study also showed that the biogas generation from poultry wastes by native and commercial farmers is below 1% and around 14%, respectively. As reported by other studies, the significant barriers may be the initial high installation cost and the lack of awareness and technical knowledge [63–65, 93]. Previous, reports have suggested that an eco-friendly appropriate waste management approach could alleviate environmental and health hazards [36, 94]. Another study reported that developing countries should consider appropriate strategies for waste management for global sustainability [95]. Optimistically, more than three-quarters of the respondents in this study think that proper waste management should be incorporated to reduce environmental degradation. Appropriate extension programs can facilitate the voluntary adoption of technology change by the farmers [96]. Previously reported data indicated that improved management skills, knowledge of waste composition, and methods are vital in adopting poultry litter as a fertilizer [97]. Governmental policies should be supportive of the change in poultry farming practices. Furthermore, half of the native poultry keepers and over three-quarters of commercial poultry farmers would consider spending a small amount of money on improving the waste management of their farms.

## Conclusion

Analysis of the overall KAP indicates the farmers’ low level of KAP toward poultry waste management. This inadequate knowledge, incorrect beliefs, and inappropriate waste management practices can negatively contribute to environmental issues. Understanding the current KAP levels, and their underlying causes could potentially be used to develop effective poultry waste management programs. Mitigating environmental pollution; poultry farming needs to adjust its management practices and production processes to be favorable for the environment.

Therefore, efforts should be made to increase farmers’ awareness and technical capacity of poultry waste management systems. Environmentally sustainable technologies and practices for the production and poultry processing activities should be adopted to reduce environmental degradation. It is also suggested that financial and technical inputs should be provided to the farmers to generate biogas and bio-fertilizer from poultry wastes. Furthermore, appropriate policies and regulations should be in place for poultry waste management.

## Authors’ Contributions

SA and TSH: Conceptualization, investigation, methodology and writing-original draft. AK, MJE, SKD, and WU: Visualization, supervision, and interpretation of data. MIZM, MB, and MRS: Supervision, methodology, and writing-review and editing. All authors have read, reviewed, and approved the final manuscript.
